# Experimental and Theoretical Investigation of External Electric-Field-Induced Crystallization of TKX-50 from Solution by Finite-Temperature String with Order Parameters as Collective Variables for Ionic Crystals

**DOI:** 10.3390/molecules29051159

**Published:** 2024-03-05

**Authors:** Fude Ren, Xiaolei Wang, Qing Zhang, Xiaojun Wang, Lingling Chang, Zhiteng Zhang

**Affiliations:** 1 School of Chemistry and Chemical Engineering, North University of China, Taiyuan 030051, China; qq886619@163.com (X.W.); qingzhang209@163.com (Q.Z.); changlingling0623@163.com (L.C.); zhitengcheer@163.com (Z.Z.); 2 Gansu Yinguang Chemical Industry Group, Baiyin 730900, China; 02056401@163.com

**Keywords:** crystallization of ionic crystal from solution, TKX-50, finite-temperature string, order parameters, external electric field, K-means clustering

## Abstract

External electric fields are an effective tool to induce phase transformations. The crystallization of ionic crystals from solution is a common phase transformation. However, understanding of mechanisms is poor at the molecular level. In this work, we carried out an experimental and theoretical investigation of the external electric-field-induced crystallization of TKX-50 from saturated formic acid solution by finite-temperature string (FTS) with order parameters (OPs) as collective variables for ionic crystals. The minimum-free-energy path was sketched by the string method in collective variables. The results show that the K-means clustering algorithm based on Euclidean distance and density weights can be used for enhanced sampling of the OPs in external electric-field-induced crystallization of ionic crystal from solution, which improves the conventional FTS. The crystallization from solution is a process of surface-mediated nucleation. The external electric field can accelerate the evolution of the string and decrease the difference in the potential of mean forces between the crystal and the transition state. Due to the significant change in OPs induced by the external electric field in nucleation, the crystalline quality was enhanced, which explains the experimental results that the external electric field enhanced the density, detonation velocity, and detonation pressure of TKX-50. This work provides an effective way to explore the crystallization of ionic crystals from solution at the molecular level, and it is useful for improving the properties of ionic crystal explosives by using external electric fields.

## 1. Introduction

Recently, external electric fields have been an effective tool to induce variations in phase transformations [1,2], with the formation of a potential new crystal phase and morphology, so as to realize control of the production process and improvements in the structures and properties of materials [3,4,5,6,7]. As mentioned by Alexander et al. [8], electric fields can reduce the nucleation time [9,10,11], increase the product yield [12,13,14], enhance overall crystal quality [10,13,15,16], and control the location of nucleation [16], the product crystal size [10,17,18], the crystal orientation [19,20,21] and polymorphism [22,23,24,25], etc.

At the molecular level, understanding the effects of electric fields on the nucleation mechanism can potentially and essentially control and enhance the crystalline product [1,5,7]. To this end, a large number of investigations have been carried out on the effects of electric fields on nucleation. In the experimental studies, Mahmood et al. reported the electric-field-induced supramolecular phase transition [5]. The electric-field-induced phase transition in perovskite relaxor ferroelectric crystals [26] and Li doping in VO_2_ film [27] were studied. For the chiral magnet Cu_2_OSeO_3_, the metastable skyrmion lattice emerged under electric fields [28]. As for the theoretical investigations conducted using ab initio density functional theory, the effects of electric fields on the phase boundaries, crystal growth rates, nucleation rates, and interfacial free energies have been studied [29,30]. The electric-field-tuned topological phase transition was investigated by first-principles calculations [31,32,33], with a change in the dipole moment [34]. Moreover, resistive switching in phase change materials [35] has recently attracted considerable attention for its application in non-volatile memory devices, and the magnetic properties have been studied [36]. Despite the extensive investigations being carried out, it remains poorly understood how the molecules aggregate and organize into a new form under external electric fields [1,3,4,5,6]. The main reason is that it exceeds the range of instrument testing for small molecules in experiments, and in theoretical simulations, the time scale is many orders of magnitude lower than that of real occurrence [37,38]. In fact, the nucleation itself is a rare event [39,40], and it is very difficult to find a set of suitable collective variables that can describe the reaction coordinate [39], which is the challenge of nucleation simulation and understanding nucleation mechanisms at the molecular level [41].

As an important rare-event method, finite-temperature string (FTS) has received great interest in exploring the phase transformation mechanisms [42,43,44,45,46,47]. With the reparameterization of the images along the path of the phase transformation [48], the minimum-free-energy path (MFEP) [49] could be obtained with the string method in collective variables (SMCV) [49] to pursue the principal curve. Furthermore, as a function of the collective motions of the system [50], the order parameters (OPs) involving only the structural parameters [39] could be used to the collective variable in describing the reaction coordinate for the phase transformation, and a corresponding MFEP could be obtained with SMCV at the molecular level. The OPs have been successfully used to monitor nucleation [39,51,52]. Moreover, K-means clustering is an algorithm by which a dataset containing *n*-dimensional vectors is clustered and divided into *k* sub-clusters by the iterations [53]. As a sampling method, it has been widely used in machine learning [54,55]. Therefore, the theoretical investigation of the phase transformation mechanism by the finite-temperature string method with order parameters as collective variables and the K-means clustering algorithm has been feasible. Recently, to improve the convergence rate of FTS with OP as collective variables for molecular crystal, we used a K-means clustering algorithm based on the Euclidean distance and sample weights to smooth the initial FTS and the minimum-free-energy path connecting between *β*-CL-20 and *ε*-CL-20 was sketched, and the free-energy profile along the path was obtained [56]. The crystallization of ionic crystals from solution is a common phase transition phenomenon, and this technique has been used abundantly in industry, with practical applications in pharmaceutical, chemical, petrochemical, food industries, and biotechnology, making it a multitrillion-dollar business [22,57,58,59,60,61]. To our knowledge, however, no theoretical investigation into the crystallization of ionic crystal from solution by the FTS method with the K-means clustering algorithm and OPs as the collective variables under the external electric field is present.

1,1’-dihydroxy-5,5’-tetrazolium dihydroxyamine salt (TKX-50) is a novel high-energy-density compound and one of the most promising third-generation energetic materials. As one of the important properties of explosives, the crystal morphology of TKX-50 has been extensively studied from theoretical simulation [62] to experiment [63] and from single component crystal [64] to co-crystal [65]. Industrial grade TKX-50 has many shortcomings, such as irregular crystal morphology, small particle size, large aspect ratio, and low intramolecular oxygen content [66], which affects its application in mixed explosives and propellants. Recrystallization from solution is a key process in the production of TKX-50 and is a common method to improve crystallization quality and application performance.

In this work, an experimental investigation of the crystallization of TKX-50 from the supersaturated formic acid solution was carried out under a direct current (DC) external electric field. To explain the experimental results, a theoretical study of the external electric-field-induced crystallization of TKX-50 from solution was presented by using the finite-temperature string method with order parameters as collective variables for ionic crystals. The local OPs of the TKX-50 crystal and the corresponding forms in the formic acid solution were constructed by the structural variables involving the bond distances, bond orientations, and relative orientations. A K-means clustering algorithm based on the dimensionally weighted Euclidean distance and sample weights was used to construct the smooth initial FTS. An MFEP involving the TKX-50 crystallization from the solution was sketched by using the SMCV method, and then the potential of mean force (PMF) was calculated under the external electric field. The essence of the crystallization of TKX-50 from the supersaturated formic acid solution was revealed. This study extends the application scope of FTS in the investigation into the crystallization from solution for the explosive ionic crystals by the K-means clustering technique with OPs as the collective variables and provides an effective way to explore the crystallization from the solution for the explosive ionic crystals at the molecular level. It is useful for further optimizing the technological process to obtain the desired explosive ionic crystals under the external electric field in the experiment.

## 2. Theory

### 2.1. Order Parameters

To gain a molecular-level understanding of the way molecules aggregate and organize themselves into crystal structures, Santiso et al. presented a method to construct OPs that involve only the structural parameters [39]. They are a set of mathematical functions containing the intermolecular distance, bond orientation, relative orientation, internal configurations, etc., and these OPs can be used to characterize the translation, vibration, and rotation of the functional groups as well as the rearrangement of molecules. Among them, the bond distance OPs, bond orientation Ops, and relative orientation OPs, represented as $\varphi_{\alpha ,i}^{d}$, $\varphi_{\alpha ,i}^{b}$, and $\varphi_{\alpha ,i}^{r}$, have been used to describe the formation of crystals in solution [39]. They are defined as follows:

(1) $$\varphi_{\alpha ,i}^{d}=\sum_{j\neq i}\frac{1}{\sqrt{2\pi }\sigma_{\alpha }}\exp\left[-\frac{{\left({\hat{r}}_{ij}-{\hat{r}}_{\alpha }\right)}^{2}}{2\sigma_{\alpha }^{2}}\right]$$

(2) $$\varphi_{\alpha ,i}^{b}=\sum \frac{1}{2\pi I_{0}\left(\eta_{\hat{r}}^{\alpha }\right)}\exp\left[\eta_{\hat{r}}^{\alpha }\cos2\left(\phi_{\hat{r}}-\phi_{\hat{r}}^{\alpha }\right)\right]$$

(3) $$\varphi_{\alpha ,i}^{r}=\sum \frac{1}{2\pi I_{0}\left(\eta_{q}^{\alpha }\right)}\exp\left[\eta_{q}^{\alpha }\cos2\left(\phi_{q}-\phi_{q}^{\alpha }\right)\right]$$

where *i* denotes the *i*th molecule; *r_ij_* is the distance between the centers-of-mass of two molecules; $\phi_{\hat{r}}$ is defined as the bond orientation from the projection of ${\hat{r}}_{ij}$ onto the absolute orientation of the *i*th molecule; $\phi_{q}$ is the relative orientation resulting from the rotation of the absolute orientation of the *i*th molecule onto *j*th molecule; and *r_α_*, $\phi_{\hat{r}}^{\alpha }$, and $\phi_{q}^{\alpha }$ are the mean center-of-mass distance, mean bond orientation, and mean relative orientation corresponding to the *α*-peak, respectively. $\sigma_{\alpha }$, $\eta_{\hat{r}}^{\alpha }$, and $\eta_{q}^{\alpha }$ are the standard deviation and concentration parameters, respectively, and *I*_0_ is the modified Bessel function of the second kind and order 0. Thus, the OPs of the bond distance combined with the bond orientation or relative orientation could be given by

(4) $$\varphi_{\alpha ,i}^{\mathrm{db}}=\frac{1}{\sqrt{2\pi }\sigma_{\alpha }}\frac{1}{2\pi I_{0}\left(\eta_{\hat{r}}^{\alpha }\right)}\sum_{j\neq i}\exp\left[-\frac{{\left({\hat{r}}_{ij}-{\hat{r}}_{\alpha }\right)}^{2}}{2\sigma_{\alpha }^{2}}\right]\exp\left[\eta_{\hat{r}}^{\alpha }\cos2\left(\phi_{\hat{r}}-\phi_{\hat{r}}^{\alpha }\right)\right]$$

(5) $$\varphi_{\alpha ,i}^{\mathrm{dr}}=\frac{1}{\sqrt{2\pi }\sigma_{\alpha }}\frac{1}{2\pi I_{0}\left(\eta_{q}^{\alpha }\right)}\sum_{j\neq i}\exp\left[-\frac{{\left({\hat{r}}_{ij}-{\hat{r}}_{\alpha }\right)}^{2}}{2\sigma_{\alpha }^{2}}\right]\exp\left[\eta_{q}^{\alpha }\cos2\left(\phi_{q}-\phi_{q}^{\alpha }\right)\right]$$

To simplify the OPs, two kinds of local OPs were often adopted [51]. One is the OP for a molecule *i* defined by summing over the different *α* peaks, and the other is defined by the averaged value within the divided cell (*C*) in the simulation box and summed over all the peaks, given by Equations (6) and (7), respectively:

(6) $$\theta_{i}^{*}=\sum_{\alpha }\varphi_{i,\alpha }^{*}$$

(7) $$\theta_{C}^{*}=\frac{1}{N_{C}}\sum_{i\in C}\sum_{\alpha }\varphi_{i,\alpha }^{*}$$

where * represents “d”, “b”, “r”, “db”, and “dr” in Equations (1)–(5), respectively.

### 2.2. Finite-Temperature String

Finite-temperature String is a technique to determine the minimum energy paths or MFEPs by evaluating locally the mean force and tensor, which accounts for the curvilinear nature of the collective variables [49,67].

Given a set of *N* collective variables used to describe the reaction of interest, *θ*(*x*) = (*θ*_1_(*x*), *θ*_2_(*x*), …, *θ_N_*(*x*)), where *x* ∈ *R^n^* are the Cartesian coordinates, the function of the free energy depending on *z* = (*z*_1_, *z*_2_, …, *z_N_*) is defined as

(8) $$F\left(z\right)=-k_{B}T\ln\left(Z^{-1}\int_{R^{n}}e^{-\beta V\left(x\right)}\delta \left(z_{1}-\theta_{1}\left(x\right)\right)\ldots \delta \left(z_{N}-\theta_{N}\left(x\right)\right)dx\right)$$

where *V*(*x*) is the potential energy, $Z=\int_{R^{n}}e^{-\beta V\left(x\right)}dx$, and *β* = 1/*k_B_T*, where *k*_B_ and *T* are the Boltzmann constant and the temperature, respectively. Note that *N* is less than the dimensionality of the full system.

For a string denoted by *z*(α, *t*) at the evolutionary time *t*, where α ∈ [0,1] is used to parametrize the string, the evolution equation involving the free-energy gradient to make it converge to MFEP could be given by

(9) $$\frac{\partial z_{i}\left(\alpha ,t\right)}{\partial t}=-\sum_{j,k=1}^{N}P_{ij}\left(\alpha ,t\right)M_{jk}\left(z\left(\alpha ,t\right)\right)\frac{\partial F\left(z\left(\alpha ,t\right)\right)}{\partial z_{k}}$$

where *P_ij_*(α, *t*) is the projector on the plane perpendicular to the path at *z*(α, *t*), *M_jk_*(*z*(α, *t*)) is the tensor, and $\frac{\partial F\left(z\left(\alpha ,t\right)\right)}{\partial z_{k}}$ (i.e., ∇F(z)) is the free-energy gradient (i.e., mean force). Given two minima of the free energy located at *z_a_* and *z_b_*, Equation (9) can be solved according to the boundary conditions *z*(0, *t*) = *z_a_* and *z*(1, *t*) = *z_b_*. As *t→*∞ the solution of (9) converges to an MFEP connecting *z_a_* and *z_b_*, and simultaneously, the tangent vector is parallel to M(z)∇F(z) at every point *z*, i.e.,

(10) $$0=\sum_{j,k=1}^{N}P_{ij}\left(\alpha \right)M_{jk}\left(z\left(\alpha \right)\right)\frac{\partial F\left(z\left(\alpha \right)\right)}{\partial z_{k}}$$

where $P_{ij}\left(\alpha \right)=\delta_{ij}-{\hat{t}}_{i}\left(\alpha \right){\hat{t}}_{j}\left(\alpha \right)$, ${\hat{t}}_{i}\left(\alpha \right)=\frac{\partial z_{i}/\partial \alpha }{|\partial z_{i}/\partial \alpha |}$, and *M_jk_*(*z*) is defined as

(11) $$M_{ij}\left(z\right)=Z^{-1}e^{\beta F\left(z\right)}\int_{R^{n}}\sum_{k=1}^{n}\frac{\partial \theta_{i}\left(x\right)}{\partial x_{k}}\frac{\partial \theta_{j}\left(x\right)}{\partial x_{k}}e^{-\beta V\left(x\right)}\delta \left(z_{1}-\theta_{1}\left(x\right)\right)\ldots \delta \left(z_{N}-\theta_{N}\left(x\right)\right)dx$$

Based on their estimators under the ergodicity with the large enough values of *k* and *T*, Equations (10) and (11) could be solved to obtain [49]:

(12) $$\frac{\partial F\left(z\right)}{\partial z_{j}}\approx \frac{k}{T}\int_{0}^{T}\left(z_{j}-\theta_{j}\left(x\left(t\right)\right)\right)dt$$

(13) $$M_{\mathrm{ij}}\left(z\right)\approx \frac{1}{T}\sum_{k=1}^{n}\int_{0}^{T}\frac{\partial \theta_{i}\left(x\left(t\right)\right)}{\partial x_{k}}\frac{\partial \theta_{j}\left(x\left(t\right)\right)}{\partial x_{k}}dt$$

Thus, Equation (8) could be solved, and a string could be evolved by the iteration of the mean force until convergence.

### 2.3. Minimum-Free-Energy Path from Finite-Temperature String

(1) Initial trajectory.

The first step for constructing FTS is to generate an initial trajectory connecting two basins. It is very difficult for the polymorphic transformation of the molecular crystals due to the size effects of the system and the dimensionality of the OP space. In most cases, either it cannot be found, or an intermediate form or a form that is not interested is present, or it leads to an irreversible or inconvergent initial string [68,69]. This can also be caused by finite size effects, i.e., if the critical nuclei are of similar size or larger than the simulation box. If system size is not an issue, one way to improve the starting string is to run an ensemble of molecular dynamics (MD) simulations starting from the point corresponding to the highest potential of mean force and neighboring points, with velocities drawn from a Maxwell–Boltzmann distribution. We can then choose the trajectories that connect two crystal-form basins and restart the string method using images from it as the initial replicas, and the corresponding initial OPs $\theta_{i,m}^{*}\left(0\right)$ and $\theta_{C,m}^{*}\left(0\right)$ can be calculated using Equations (6) and (7) by an MD simulation.

(2) K-means clustering to obtain a smooth initial string.

In order to obtain a smooth initial string, a K-means clustering algorithm can be adopted. It consists of four steps:

*Step 1. Restricting sampling*. In each independent space around each given representative point, the samples can be taken according to the following harmonic functions:

(14) $$\psi \left(\theta_{C}^{d,b}\right)=\frac{k_{d}}{2}{\left[\theta_{C,m}^{d}-\theta_{C,m}^{d}\left(0\right)\right]}^{2}+\frac{k_{b}}{2}{\left[\theta_{C,m}^{b}-\theta_{C,m}^{b}\left(0\right)\right]}^{2}$$

(15) $$\psi \left(\theta_{C}^{d,r}\right)=\frac{k_{d}}{2}{\left[\theta_{C,m}^{d}-\theta_{C,m}^{d}\left(0\right)\right]}^{2}+\frac{k_{r}}{2}{\left[\theta_{C,m}^{r}-\theta_{C,m}^{r}\left(0\right)\right]}^{2}$$

where $k_{d}$, $k_{b}$, and $k_{r}$ are the spring coefficients. In this way, each simulation was localized in the independent space determined by each of the representative replicas.

*Step 2. K-means clustering*. Assumed that there is always a clustering center with the maximum weight in each independent space, and the OPs corresponding to the clustering center with the maximum weight are different in the different replicas. Obviously, such a center could be regarded as a locally important sampling point, which, to some extent, shows the evolution direction of the representative points along the string. Therefore, the application of the clustering algorithm can not only find the clustering center with the largest weight to solve the problem of distinguishing highly similar OPs in the adjacent replicas but also accelerate the convergence rate.

For the clustering algorithm, the dimensionally weighted Euclidean distance was used to measure the distance between sample points; after calculating the density and weight of all samples, the point with the highest weight can be selected in each independent sampling space. The weight *w_i_* is given by

(16) $$\omega_{i}=\rho_{i}\times s_{i}\times \frac{1}{a_{i}}$$

where *ρ_i_*, *s_i_*, and *a_i_* represent the density of sample point *i* (i.e., the number of points *i* within a certain range), the distance between different clusters or datasets, and the distance between different sample points in the same cluster, respectively. They can be calculated by Equations (17) and (18) as follows:

(17) $$\rho_{i}=\sum_{j=1}^{n}f\left[d_{\omega }\left(x_{i},x_{j}\right)-\frac{1}{C_{n}^{2}}\sum_{i=1}^{n}\sum_{j=i+1}^{n}d_{\omega }\left(x_{i},x_{j}\right)\right]$$

(18) $$s_{i}=\{\begin{array}{c} \min\left(d_{\omega }\left(x_{i},x_{j}\right)\right),\rho_{j}<\rho_{i}\\ \max\left(d_{\omega }\left(x_{i},x_{j}\right)\right),\rho_{j}\leq \rho_{i} \end{array}$$

(19) $$a_{i}=\frac{2}{\rho_{i}\left(\rho_{i}-1\right)}\sum_{i=1}^{\rho_{i}}\sum_{j=i+1}^{\rho_{i}}d_{\omega }\left(x_{i},x_{j}\right)$$

where $d_{\omega }\left(x_{i},x_{j}\right)$ is the Euclidean distance, and $f\left(x\right)=\{\begin{array}{c} 1,x<0\\ 0,x\geq 0 \end{array}$. These adjacent clustering centers with the maximum weights can be connected to form a temporary string.

*Step 3. Searching for successive strings by smooth function*. In order to obtain a successive string in the collective coordinate space, the smooth function was defined as follows:

(20) $$S^{*}=\sum_{m=2}^{L-1}\exp[\theta_{C,m}^{*}-\theta_{C,average\left(m-1,m+1\right)}^{*}]$$

where $\theta_{C}$_,_$averge\left(m-1,m+1\right)=\left(\theta_{C,m-1}+\theta_{C,m+1}\right)/2$. The smoothness of the temporary string was calculated by the Monte Carlo method. Firstly, the smoothing score of the current temporary string was calculated. Then, in the same space, another clustering center, where the weight difference from the first clustering center is small, was selected, and the smoothing score was recalculated. According to the new smoothing score, whether to accept the new change was determined according to the metropolis criterion. Finally, a string with the lowest smoothing score was obtained.

*Step 4. Calculating* $z_{C,m}^{*}$. Based on the optimization string, OPs of the *m*th replica were calculated as the initial OPs to evolve string, represented by $z_{C,m}^{*}$. For comparison, the values of $z_{C,m}^{*}$ were also directly calculated without K-means clustering, i.e., average-based sampling.

(3) Determining the MFEP using the SMCV method.

The MFEPs can be determined with the SMCV method by the following steps:

*Step 1. Estimating* $\nabla_{z}F(z_{C,m}^{*})$ *and* $M(z_{C,m}^{*})$. Based on the initial string with or without clustering, the free-energy gradient $\nabla_{z}F(z_{C,m}^{*})$ and metric tensor $M(z_{C,m}^{*})$ can be estimated by using Equations (12) and (13), respectively.

*Step 2. Calculating a new target OPs* $z_{C,m(\mathrm{new})}^{*}$. Following Maragliano [70], $z_{C,m(\mathrm{new})}^{*}$ was calculated by the string evolution equation,

(21) $$z_{C,m(\mathrm{new})}^{*}=z_{C,m}^{*}-\Delta \tau M\left(z_{C,m}^{*}\right)\nabla F\left(z_{C,m}^{*}\right)$$

*Step 3. Reparameterizing*. Interpolate a curve *z*(α) through $z_{C,m(\mathrm{new})}^{*}$ by the b-spline fitting method and compute the new targets OPs (represented as $z_{C,m+1}^{*}$) by interpolating to make the arc length at a constant between the consecutive replicas.

*Step 4. Obtaining MFEP under convergence*. Compute the difference between $z_{C,m+1}^{*}$ and $z_{C,m}^{*}$. If it is small enough, the string is converged, and the MFEP is obtained by the converged target OPs; otherwise, go back to *Step 1*, and the iterative process is continued until it converges.

*Step 5. Calculating local OPs and PMF.* Based on the final converged string, the local OPs and PMF were calculated.

## 3. Results and Discussion

### 3.1. Crystallization of TKX-50 by DC External Electric Field

**Figure 1:**
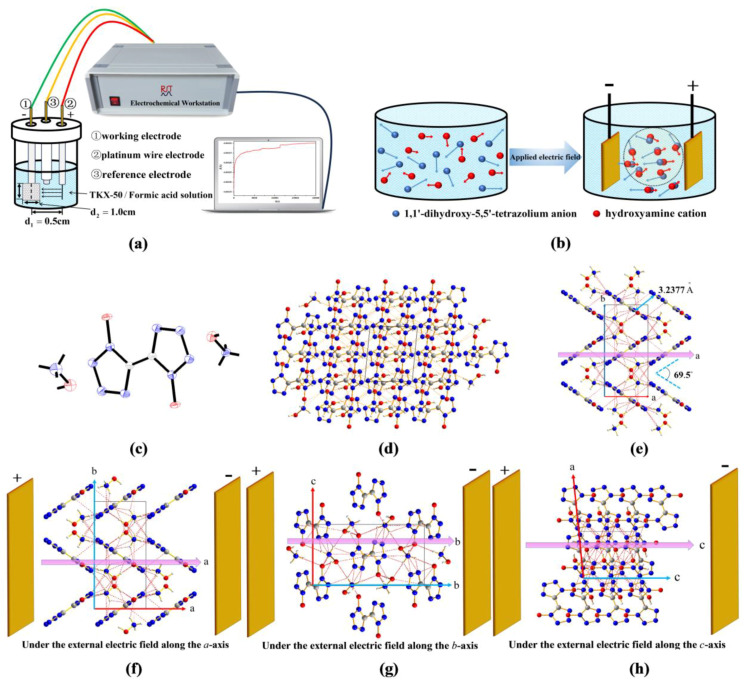
Experimental setup of DC external electric field and crystallization of TKX-50. (**a**): Experimental setup; (**b**): Schematic diagram of anion motion in an external electric field; (**c**):Molecular structure of TKX-50 obtained from experiment; (**d**): Molecular stacking in crystals; (**e**): Perspective of molecular stacking from the BC plane direction of the crystal (**f**–**h**): The directions of the external electric fields along the three axes. Note: In (**f**–**h**), a, b, and c mean the a-axis, b-axis, and c-axis, respectively.

The experiment on the crystallization of TKX-50 from the supersaturated formic acid solution was carried out under the DC external electric field. The experimental setup is in Figure 1, and the molecular structure of TKX-50 and crystal data are shown in Figure S1 and Tables S1–S10.

Although the space group of the TKX-50 crystal was not affected by the external electric field, and the crystalline structure of TKX-50 has a monoclinic crystalline structure with space group P2_1_/c, the crystal data and structural parameters are changed, obviously. In particular, the volume decreased, and the density increased by 0.1 g/cm^3^. For the explosive, the higher the density, the higher the detonation velocity and pressure become. Therefore, the introduction of the external electric field into the explosive system is beneficial for improving the performance of explosives. Moreover, from Tables S3 and S8, it can be seen that the bond length of C–N on the TKX-50 tetrazole ring is shortened by an average of 0.04 Å under the external electric field, suggesting that the external electric field enhances the stability of TKX-50.

**Figure 2:**
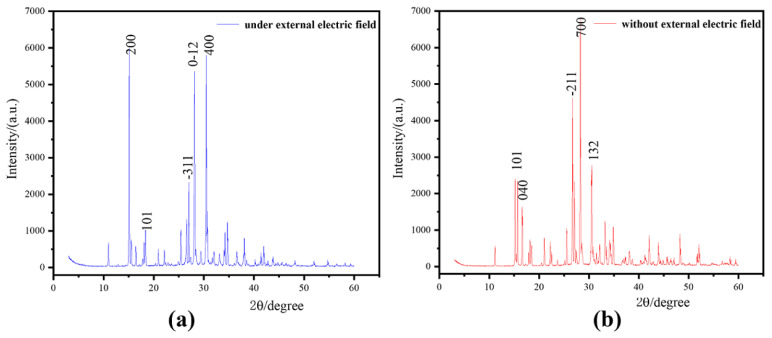
XRD powder diffraction of TKX-50. (**a**): XRD under external electric field; (**b**): XRD without external electric field.

The X-ray powder diffraction is shown in Figure 2. Without the external electric field, TKX-50 exhibits the characteristic diffraction peaks at 15.03, 15.46, 26.12, 27.00, and 31.15. Under the external electric field, the diffraction peaks undergo changes, appearing at positions 15.01, 16.27, 26.54, 27.05, and 30.89. In particular, compared with the diffraction peaks without the electric field, the non-diffraction characteristic peaks under the electric field are fewer, indicating that the crystalline quality and purity of TKX-50 are enhanced under the external electric field.

### 3.2. MD Simulation on Crystallization of TKX-50 without External Electric Field

#### 3.2.1. Peaks in Pair Distribution Function

The peaks in the pair distribution functions for the reference structure of the TKX-50 crystal and the corresponding average peak locations and concentration parameters at 300 K with a cutoff of 10.0 Å are given in Table 1. The peak locations are very close to the experimental results. The smaller standard deviation and larger concentration parameter display the localization characteristics of the average centroid distance, bond orientation, and relative orientation distribution.

**Figure 3:**
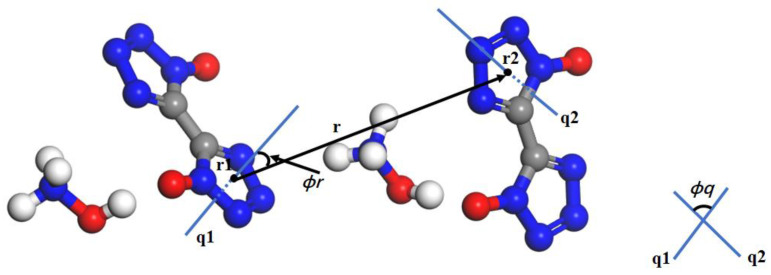
Construction of OPs for TKX-50. Red, blue, gray, and white represent O, N, C, and H atoms, respectively. The vector ***r*** joins the center of mass of the two molecules. The direction of the axis across the center of the ring can be used as an approximate measure of the absolute orientation (*q*_1_ or *q*_2_). The bond orientation $\phi_{\hat{r}}$ is defined as the projection of ${\hat{r}}_{ij}$ onto *q*_1_, and the relative orientation $\phi_{q}$ shows the rotation of *q*_1_ onto *q*_2_.

**Figure 4:**
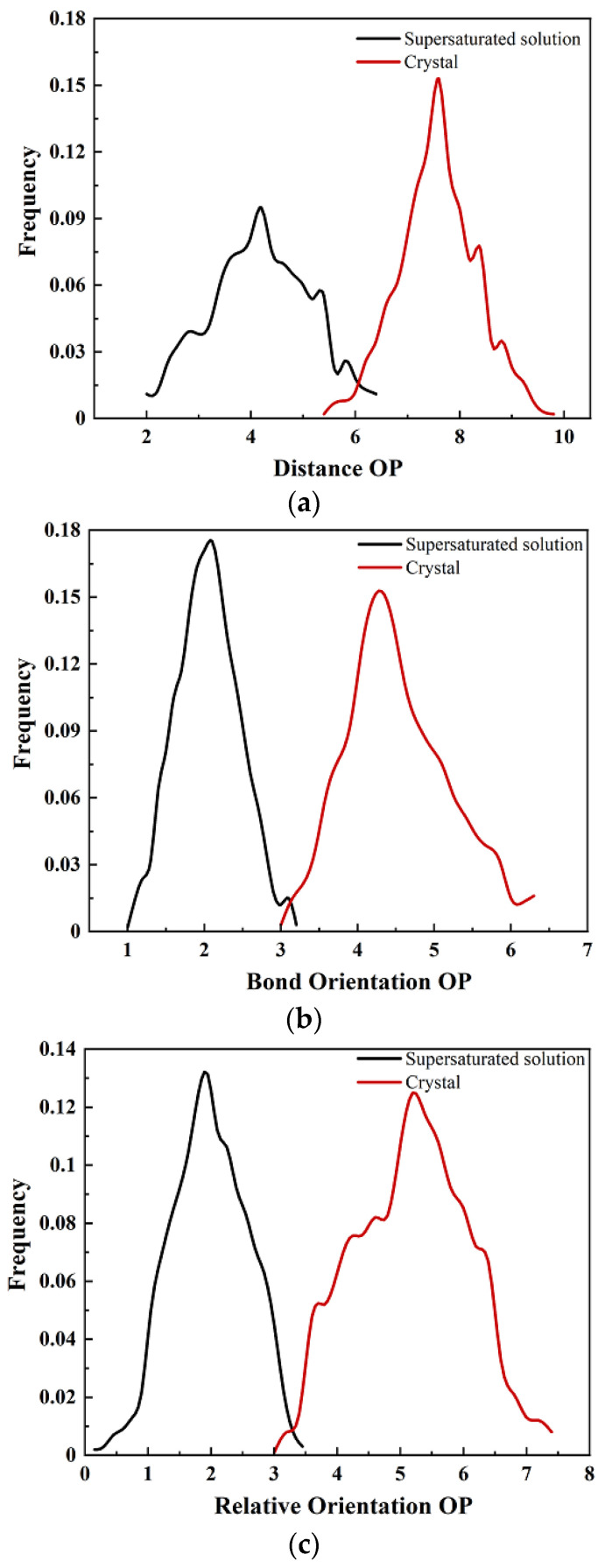
The distribution of (**a**) distance, (**b**) bond orientation, and (**c**) relative orientation OPs for TKX-50 crystal and the corresponding forms in the formic acid solution. These distributions were obtained by considering 2.5 ns MD simulations in the *NPT* ensemble from the averaged values within the divided cells with the 3 × 3 × 3 grid for each of the systems with 216 TKX-50 molecules.

For the homogeneous nucleation of crystals of the ionic liquid [dmim^+^][Cl^−^] from its supercooled liquid phase, two [dmim^+^][Cl^−^] ion pairs show the variables relevant for the construction of OPs, which are based on the [dmim^+^] cations. The vector *r* joins the center of mass of the two cations. The vector normal to the plane of the imidazolium ring gives the absolute orientation *q* of each of the cations [71]. Similarly, according to the construction method of OPs by Santiso et al. [39], the vector *r* joins the center of mass of two tetrazolium anion rings, and the absolute orientation *q* was given by two anions. The bond orientation $\phi_{\hat{r}}$ and the relative orientation $\phi_{q}$ were also given by two anions; see “Section 4.2”. Thus, the local OPs of the TKX-50 crystal and the corresponding forms in the formic acid solution were constructed by the structural variables involving the bond distances, bond orientations, and relative orientations (see Figure 3). After obtaining the maximum-likelihood estimators, they were calculated (see Figure 4). Although three OPs of the TKX-50 crystal phase and the corresponding forms in the formic acid solution overlap slightly, the peaks exhibit significantly different values. Therefore, any of the OPs can serve as a good metric to detect the orders of the crystalline and the corresponding forms in the formic acid solution. In this work, we chose the bond orientation OPs and relative orientation OPs during all our simulations.

#### 3.2.2. Convergence of FTS and K-Means Clustering

**Figure 5:**
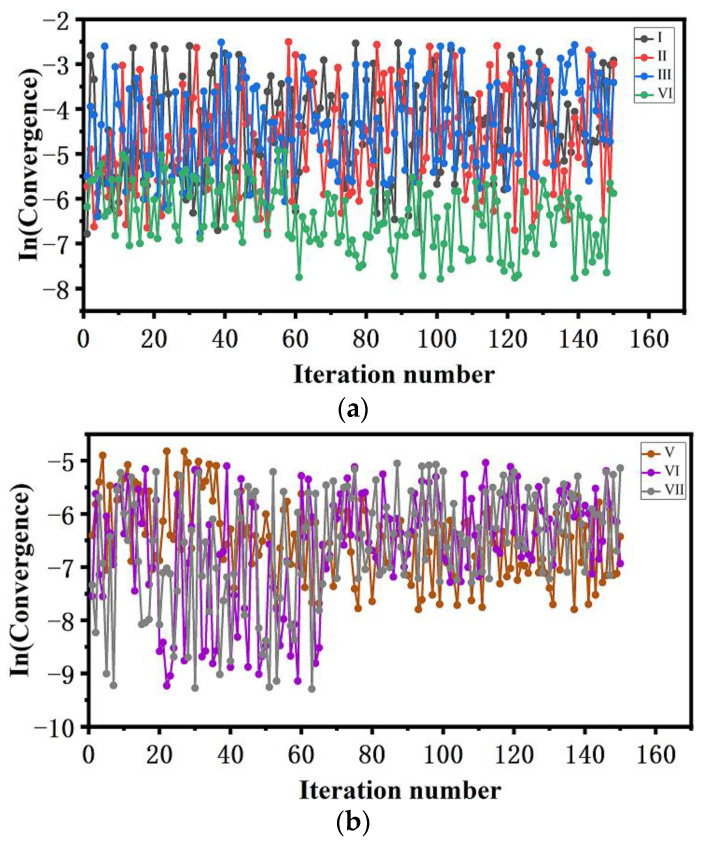
Convergence of collective variables during the evolution of the string. (I) and (II) correspond to $\phi_{C}^{db}$ with K-means and without K-means clustering, respectively; (III) and (IV) correspond to $\phi_{C}^{dr}$ without K-means clustering and with K-means clustering, respectively. (V), (VI), and (VII) correspond to $\phi_{C}^{dr}$ with K-means clustering for the convergence of collective variables under an external electric field with the strength of 51.40 × 10^8^ V/m along the *a*-, *b*-, and *c*-axes, respectively. (**a**) Convergence of collective variables without external electric field; (**b**) convergence of collective variables under external electric field.

According to the trajectories from the simulation of 3 × 5 ns for 21 independent spaces, the average sampling and K-means clustering of OPs for bond orientation and relative orientation were performed, as shown in Figure 5. It was found that for $\phi_{C}^{db}$, the ln(Convergence) values are in the range of −5.5~−2.5 after about 20 iterations, indicating that there has been convergence. However, whether for the ln(Convergence) values from the average sampling or the K-means clustering, the changes are still uncertain and remain oscillating after 150 iterations. In this case, the simulation may cover adjacent spaces along the transition path, leading to an uneven initial string connecting adjacent points. For the non-smooth string, there are always some independent sampling spaces that are not perpendicular to the current string, leading to confusion in the evolution direction of the string.

As for $\phi_{C}^{dr}$, compared to the ln(Convergence) values from the average sampling, those from the K-means clustering have decreased after 60 iterations. This shows that the application of the K-means clustering algorithm can not only quickly find the cluster center with the highest weight and solve the differentiation of highly similar OPs in adjacent replicas but also accelerate the convergence speed of strings. Generally, the K-means clustering algorithm combining dimensionally weighted Euclidean distance with density weight has a strong outlier interference ability, which can effectively avoid local optima and quickly converge to global optima to a certain extent. These are in agreement with our recent investigation [72].

#### 3.2.3. Minimum-Free-Energy Path

PMF is the line integral of the restraint force along the path, and it shows the MFEP as the maximum-likelihood path in the space of collective variables along FTS [67,70].

**Figure 6:**
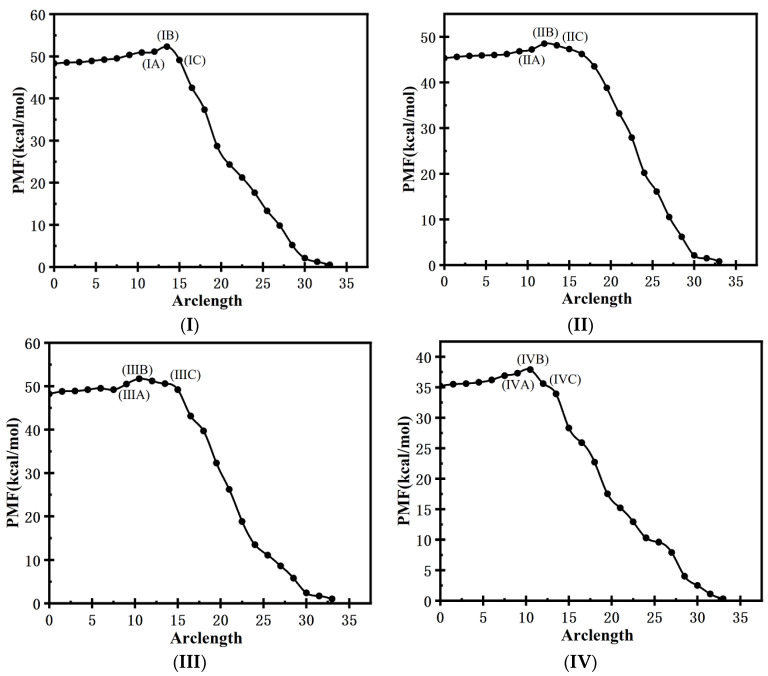

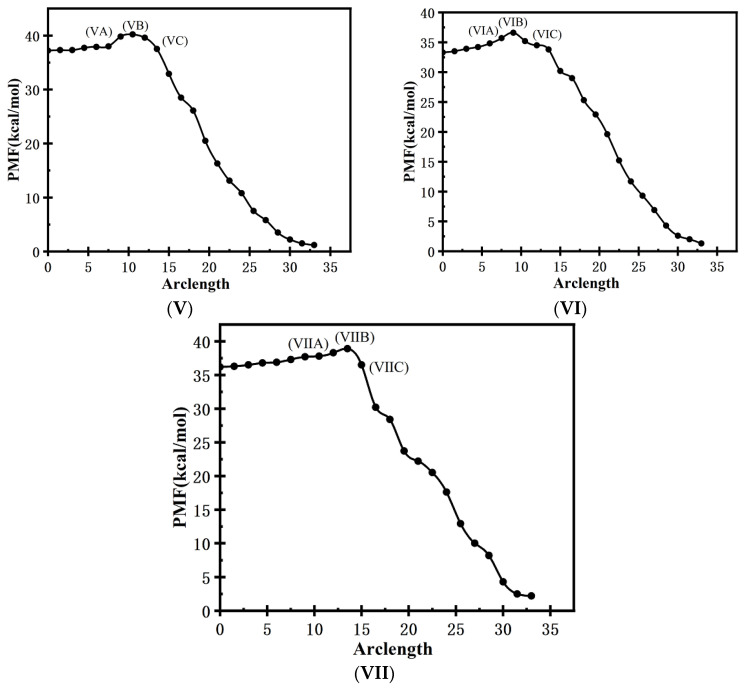
PMF as a function of arclength along the FTS path for the converged string obtained from the SMCV. The initial point at arclength zero is the supersaturated formic acid solution of TKX-50, and the endpoint is TKX-50 crystal. (**I**,**II**) correspond to the FTS path obtained from $\phi_{C}^{db}$ with K-means and without K-means clustering; (**III**,**IV**) correspond to the FTS path obtained from $\phi_{C}^{dr}$ without K-means clustering and with K-means clustering; (**V**–**VII**) correspond to the FTS path obtained from $\phi_{C}^{dr}$ with K-means clustering for the convergence of collective variables under external electric field with the strength of 51.40 × 10^8^ V/m along the *a*-, *b*-, and *c*-axes, respectively. In every image, B means the transition state, and A and C mean the state similar to the solution and the crystal, respectively.

The convergent FTS path from the TKX-50 crystal (isolated from the formic acid phase) to its supersaturated formic acid solution was obtained using the SMCV method. Figure 6I,II show the PMF curves as the function of the arclength along the converged FTS paths corresponding to the $\phi_{C}^{db}$ as the collective variables by K-means clustering and without K-means clustering, respectively. (III) and (IV) correspond to the FTS path obtained from $\phi_{C}^{dr}$ without K-means clustering and with K-means clustering. The difference in PMF between the supersaturated formic acid solution and the transition state is small, with the value less than 4.0 kJ∙mol^−1^ in all the cases, while the difference in PMF between the TKX-50 crystal and the transition state is large, close to 50.0 kJ∙mol^−1^ from $\phi_{C}^{db}$ in two cases and $\phi_{C}^{dr}$ as the collective variables by K-means clustering, and about 40.0 kJ∙mol^−1^ from $\phi_{C}^{dr}$ without K-means clustering. These results show that, at 300 K, the solubility of TKX-50 in formic acid is not high, while its saturated solution is prone to crystallization. This is in agreement with our experimental result (0.5 g TKX-50 is dissolved in 45 mL formic acid in this work). Note that due to PMF corresponding to the dimensions (3 × 3 × 3), instead of one-dimensional reaction coordinates, the values of PMF are higher than the real free-energy barrier. Moreover, the differences in PMF between the transition state and TKX-50 are close to each other, and those between the transition state and the supersaturated formic acid solution are also close to each other. This indicates that the difference is not related to the type of OPs or whether it is K-means clustering, as is in agreement with our previous investigation [56].

**Figure 7:**
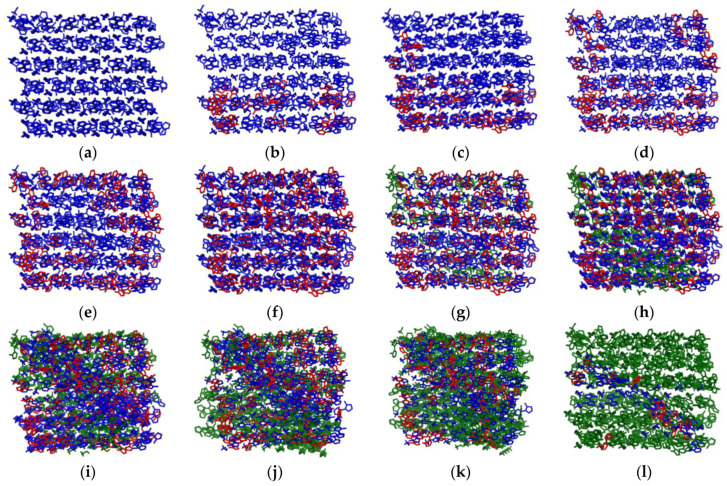
Changes in local order parameters on the FTS path shown by the key snapshots. Along the (**a**–**l**), the type of molecule gradually changes from the “all blue” initial conformation, the formation of molecular clusters of which “Scattered green areas are surrounded by blue areas”, to gradually increasing “sporadic green” molecular clusters, and then to the “blue being engulfed by green” conformation. (**a**–**l**) **mean the several** key snapshots from crystal to supersaturated formic acid solution of TKX-50 (the molecules of formic acid were deleted).

In order to gain further mechanistic insights into the crystallization of TKX-50 from the supersaturated formic acid solution, the local OPs of TKX-50 were shown for the key snapshots at the points of the PFM curve along the trajectory going from crystal to solution (see Figure 7). The molecules that can be regarded as existing in the crystal were labeled in blue with the values of $\phi_{C}^{db}$ > ~140 or $\phi_{C}^{dr}$ > ~160, and the green molecules were regarded as TKX-50 in solution with the values of $\phi_{C}^{db}$ < ~50 or $\phi_{C}^{dr}$ < ~40, whereas the wireframe molecules in red formed the interface between crystal and solution with the values in the range of 50~140 or 40~160 for s$\phi_{C}^{db}$ and $\phi_{C}^{dr}$, respectively.

From Figure 7, it can be seen that along the (a) → (b) → … → (l), the type of molecule gradually changes from the “all blue” initial conformation, the formation of molecular clusters of which “Scattered green areas are surrounded by blue areas” (interface induction), to gradually increasing “sporadic green” molecular clusters (i.e., local initiation, multinuclear asynchronous generation, and increase), and then to the “blue being engulfed by green” conformation. In most cases, OPs show a central region with the TKX-50 structure surrounded by molecules with intermediate values of OPs corresponding to the surface of the crystal. This indicates that similar to the polymorphic transformation [56,72], the crystallization from solution is also an interface-induced and locally initiated development process.

### 3.3. MD Simulation of Crystallization of TKX-50 under the External Electric Fields

The average peak locations and concentration parameters under the external electric fields with the strength of 51.40 × 10^8^ V/m are shown in Table 2. Compared with the values without the external electric fields, both the peak values and the concentration parameters of the pair distribution functions for TKX-50 crystal change significantly, especially for them under the external electric field along the direction of the *c*-axis. As expected, due to two hydroxyamine groups having a positive charge and the dihydroxy-5,5’-tetrazolium group having a negative charge, they will undergo significant displacement under the external electric field, resulting in a significant change in the peak values and the concentration parameters, as shown in Figure 1c. Most of the directions of the positive and negative charges for hydroxylamine and dihydroxy-5,5’-tetrazolium groups are exactly along the direction of the *c*-axis of the crystal (see Figure 1h), so the changes are more significant along the *c*-axis. Moreover, in most cases, the values of $\phi_{q}$ have decreased while those of $\phi_{\hat{r}}^{\alpha }$ increased, showing that the π∙∙∙π stacking is more significant under the external electric field.

Figure 5b gives the convergence of $\phi_{C}^{dr}$ under an external electric field with a strength of 51.40 × 10^8^ V/m by K-means clustering. Compared with those without the external electric field (see (IV)), the values of the ln(Convergence) of the relative orientation OPs are decreased greatly, and there is convergence after 60 iterations in all cases. This shows that the external electric field can accelerate the evolution of the string. In 2017, Jha et al. found that external electric fields can obviously increase the nucleation rate [59]. Koizumi et al. also observed the same phenomenon [73]. These results confirmed that, shown by the accelerated evolution of the string, the external electric field can accelerate the nucleation rate.

For the $\phi_{C}^{dr}$ as the collective variables, similar to the cases without an electric field, the difference in PMF between the supersaturated formic acid solution and the transition state is also small, with the value less than 3.5 kJ∙mol^−1^ under the external electric field. The difference in PMF between the TKX-50 crystal and the transition state is less than 40.0 kJ∙mol^−1^ under the external electric fields with a strength of 51.40 × 10^8^ V/m along the *a*-, *b*-, and *c*-axes. These values are decreased in comparison with those in no field. Moreover, the fields parallel to the *b*- and *c*-axes affect the difference in PMF between the TKX-50 crystal and the transition state less than those parallel to the *a*-axis. The crystallization of TKX-50 from the formic acid solution is mainly closely related to the intermolecular interaction. From Figure 1f–h, along the *a-*axis, the strong H-bonding interactions are formed. They are strengthened by the increased dipole moments more significantly under the external electric fields, leading to a large value of the PMF between the TKX-50 crystal and the transition state along the *a*-axis. The value of the PMF between them is the smallest along the *b*-axis. From Figure 1f–h, the intermolecular π∙∙∙π stackings are formed along the *b*-axis. Due to the weaker π∙∙∙π stacking than H-bonding interaction, the more notable change will occur under the external electric field along the *b*-axis, leading to the low energy of the transition state and thus the smaller difference in PMF. Therefore, applying an external electric field along the *b*-axis direction has more practical value for achieving the crystallization of TKX-50 from solution.

**Figure 8:**
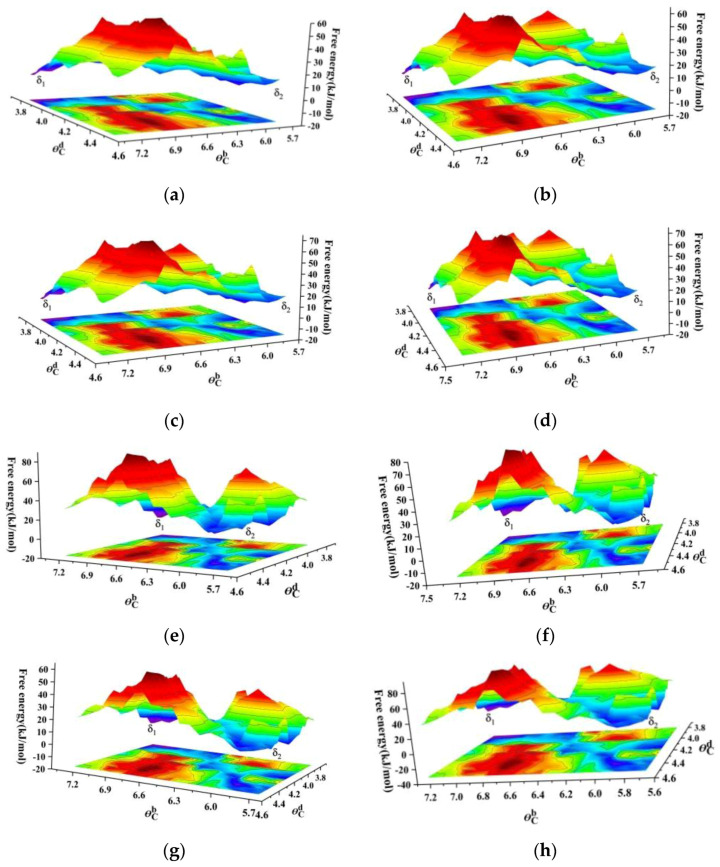
Three-dimensional surface plots of the free-energy landscape obtained from $\theta_{C}^{d}$ and $\theta_{C}^{b}$ as the collective variables for the crystallization of TKX-50 from the supersaturated formic acid solution under the external electric fields along the *b*-axis: (**a**) 10.28 × 10^8^ V/m; (**b**) 20.56 × 10^8^ V/m; (**c**) 30.84 × 10^8^ V/m; (**d**) 41.12 × 10^8^ V/m; (**e**) 51.40 × 10^8^ V/m; (**f**) 61.68 × 10^8^ V/m; (**g**) 71.96 × 10^8^ V/m; (**h**) 82.24 × 10^8^ V/m.

In order to further verify the influence of the external electric field along the *b*-axis direction on the crystallization of TKX-50 from the supersaturated formic acid solution, the external electric fields were added with the field strengths of 0.514 × 10^8^, 5.14 × 10^8^, and 10.28 × 10^8^~102.80 × 10^8^ V/m with a step of 10.28 × 10^8^ V/m. The values of the difference in PMF between the TKX-50 crystal and the transition state were calculated to be 51.3, 48.2, 37.6, 42.5, 40.1, 38.3, 36.6, 35.3, 39.7, 42.5, 46.8, and 32.5 kJ/mol. The corresponding 3D surface plots of the free-energy landscape obtained from $\theta_{C}^{d}$ and $\theta_{C}^{b}$ as the collective variables are shown in Figure 8. The influence of the external electric field on PMF has no regular pattern in values. For the former, a good linear result (*R*^2^ = 0.9882) was found, showing that the external electric fields (*F*) have an obvious influence on the polymorphic transformation from *o-*TNT to *m-*TNT. For the latter, this relationship was not discovered from the above values of the difference in PMF between the TKX-50 crystal and the transition state. As mentioned above, we only found that the values of the difference in PMF are decreased in comparison with those in no field. It is well-known that the movement of ions in an external electric field is complex, and in the process of the crystallization from solution, this movement pattern may be more complex, leading to irregular changes in the difference in PMF. However, one conclusion can be confirmed that the external electric fields can reduce the difference in PMF and thus accelerate the nucleation rate, as is also confirmed by the experimental result [59].

### 3.4. Detonation Performance

According to the densities of TKX-50 from the experiments (1.920 g/cm^3^, see Table S1 without field vs. 1.933 g/cm^3^, see Table S6 under external electric field), the calculated detonation velocity (*V_D_*) and detonation pressure (*P_D_*) were 9604 m/s and 42.49 GPa in no field, and 9650 m/s and 43.07 GPa with an external electric field, respectively. The results of *V_D_* and *P_D_* without field are consistent with those in Reference [74]. Compared with the values without the external electric fields, the values of *V_D_* and *P_D_* under the external electric field were increased by 0.48% and 1.37%, respectively. This indicates that the external electric field can enhance the detonation performance of TKX-50.

In summary, according to the experimental results, the structural parameters of the TKX-50 crystal were changed under the external electric field, leading to increased density, detonation velocity, and detonation pressure. From the MD simulation, the K-means clustering algorithm based on Euclidean distance and density weights can be used for enhanced sampling of the OPs in external electric-field-induced crystallization of ionic crystals from solution, which can improve the conventional FTS. The crystallization from solution is a process of surface-mediated nucleation. The external electric fields can improve the conventional FTS and decrease the difference in PMF between the crystal and the transition state. This is attributed to the significant changes in the OPs under the external electric field, which leads to the enhanced crystalline quality and purity of TKX-50 observed from the experiment. This suggests that the process of the crystallization of TKX-50 from solution can be controlled by changing the external electric fields to enhance the crystalline quality and purity.

## 4. Experiment and MD Simulation Details

### 4.1. Experiment

An electrochemical workstation was used to recrystallize TKX-50 under the external electric field. Dissolve TKX-50 (0.5 g, purity: 99.2%) in formic acid (45 mL, concentration: 88%) and prepare it as a supersaturated solution. The distance between the working electrode and the auxiliary electrode is 0.5 cm. The working electrode is a square platinum plate electrode with a side length of 1.0 cm, and a constant voltage of 2V was used.

The TKX-50 crystals with the dimensions of 0.16 mm × 0.09 mm × 0.08 mm obtained without the external electric field and those with the dimensions of 0.12 mm × 0.07 mm × 0.05 mm obtained under the external electric field were selected. They were placed on a Bruker D8 VENTURE single-crystal diffractometer. MoK*α* Ray (λ = 0.71073 Å) monochromated by graphite monochromator was as the X-ray source, with ω-scanning method within a certain scanning range *θ*.

### 4.2. MD Simulation Details

Based on our experimental structure (see Table S1), the TKX-50 crystal structure was constructed with the size of 6 × 6 × 6 (216 molecules). Based on the molecular structure and strategy of the OP construction for molecular crystal [39], a set of OPs for TKX-50 was built with the pair distribution function (see Figure 2).

The crystallization of TKX-50 from solution involves mainly the disruption of the cohesive forces in the crystal lattice. Due to the insignificant –NH_3_ rotation, the internal degrees of freedom of conformational transformation can be ignored. Based on molecular symmetry and point molecule representation [69], The vector *r* joins the center of mass of the two tetrazolium rings. The vector normal to the plane of the tetrazolium ring gives the absolute orientation *q* of each of the tetrazolium anions. The bond orientation $\phi_{\hat{r}}$ is given by the angle formed by the vectors *r* and *q*1, and the relative orientation $\phi_{q}$ is given by the angles between the vectors *q*1 and *q*2, as shown in Figure 2.

In order to construct the above OPs, a short (500 ps) MD simulation was first carried out for the structure of TKX-50 from the experimental crystal and its supersaturated formic acid solution at 300 K and 1 bar. After equilibration, the structures were annealed to zero temperature at constant volume and relaxed by energy minimization. Then, an MD simulation (2.5 ns) was carried out at 300 K and 1 bar. The trajectory was used to obtain maximum-likelihood estimators of parameters in Equations (6) and (7) in the case of the simulated box with the 3 × 3 × 3 grid.

A 1.0 ns MD simulation was carried out on the system with the 216 TKX-50 molecules at 300 K and 1 bar. The long-range electrostatics were handled using the particle mesh Ewald method [75] with a cutoff of 12 Å, and a Langevin thermostat with an oscillation frequency of 25 ps^−1^ was introduced for the temperature control. The Nosé–Hoover–Langevin piston with a damping time of 50 fs was used to control the pressure. The hydrogen bond lengths were constrained with the LINCS algorithm [76]. Periodic boundary conditions were applied in all directions. After equilibrium, annealing treatment was carried out at a constant volume to zero temperature, and energy minimization relaxation was carried out. Then, the temperature was raised to 300 K, and a 2.5 ns MD simulation was carried out at a constant volume with a time step of 0.5 fs. Based on the obtained trajectory, the parameters of Equations (1)–(7) were calculated using the maximum-likelihood estimation method, and the initial order parameters were determined.

The external electric fields with a strength of 51.40 × 10^8^ V/m were added along the positive direction of the *a*-, *b*-, and *c*-axes. For the *c*-axis, the field strengths are 0.00, 0.514 × 10^8^, 5.14 × 10^8^, and 10.28 × 10^8^~102.80 × 10^8^ V/m with a step of 10.28 × 10^8^ V/m were also applied. The MD simulation of the structural optimization was carried out by Materials Studio 5.0 software package with COMPASS force field.

For the MD simulation of FTS evolution, the ensemble remains at NPT (300 K and 1 bar). For each initial configuration, we choose the configuration that is closest to the new target OP from the previous step. To maintain the stability of the simulation, the center of the OP constraint is moved from the corresponding value in the previous step to a new value in the previous period of time. Average the constraint force for each step in the later period, and the initial velocity is randomly assigned from the Maxwell–Boltzmann distribution of the temperature at which it is located. The CHARMM22 force field was used for the melting of benzene, and the calculated results were consistent with experimental values [69]. Therefore, the CHARMM22 force field was selected. All the calculations were carried out by a modified version of the NAMD 2.14 software package [77] that included implementations of the OPs and the SMCV. All the simulations were performed in the NPT ensemble with P = 1 bar and T = 300 K.

### 4.3. Detonation Performance Calculations

The detonation velocity (*V_D_*) and detonation pressure (*P_D_*) can be evaluated by Kamlet approximation, as shown by Equation (22) [78]:

(22) $$\begin{array}{l} V_{D}=1.01{\left(N^{2}\bar{M}Q_{D}\right)}^{\frac{1}{4}}\left(1+1.30d\right)\\ P_{D}=1.558{\left(N^{2}\bar{M}Q_{D}\right)}^{\frac{1}{2}}d^{2} \end{array}$$

where *N* is the moles of gaseous detonation products per gram of explosive, $\bar{M}$ is the average molecular weight of gaseous products, and *Q_D_* and *d* are the heat of detonation reaction and density of explosives, respectively.

## 5. Conclusions

In this work, an experimental investigation on the crystallization of TKX-50 from the supersaturated formic acid solution was carried out under the DC external electric field. To explain the experimental results, a theoretical study was presented by FTS with the local OPs as collective variables for ionic crystals. A K-means clustering algorithm based on the dimensionally weighted Euclidean distance and sample weights was used to smooth the FTS. The MFEP involving the crystallization of TKX-50 from solution was sketched by the SMCV method. The following conclusions were drawn from this study:

(1) From the experiment, the external electric field enhanced the density, detonation velocity, and detonation pressure of TKX-50.

(2) The K-means clustering algorithm based on Euclidean distance and density weights can be used for enhanced sampling of the OPs in the external electric-field-induced crystallization of the TKX-50 ionic crystal from solution, which can improve the conventional FTS.

(3) The crystallization of TKX-50 from the formic acid solution under the external electric field could be regarded as a process of surface-mediated nucleation.

(4) Due to the significant changes in OPs, the external electric fields accelerate the evolution of the string and decrease the difference in PMF between the crystal and the transition state. The process of the crystallization of TKX-50 from solution can be controlled by changing the external electric fields with enhanced crystalline quality and purity.

This work provides an effective way to explore the crystallization of ionic crystals from solution at the molecular level, and it is useful for realizing the control of the production process and the improvement in the properties of ionic crystal explosives by using external electric fields.

## Figures and Tables

**Table 1:** Average peak locations and concentration parameters for TKX-50 crystal at 300 K ^a^.

*r* (Å)	1/*σ*^2^ (Å^−1^)	$\phi_{\hat{r}}^{\alpha }$ (°)	$\eta_{\hat{r}}^{\alpha }$	$\phi_{q}$ (°)	$\eta_{q}^{\alpha }$
5.53 (5.46)	28.56	11.53 (12.68)	31.25	75.31 (69.68)	11.26
7.56 (7.62)	5.19	69.82 (67.53)	28.62	43.26 (44.52)	23.18
8.69 (8.43)	16.40	78.93 (83.26)	10.63	14.83 (16.83)	17.53

^a^ The values in parentheses are the experimental results.

**Table 2:** Average peak locations and concentration parameters for the TKX-50 crystal at 300 K under the external electric field with the strength of 51.40 × 10^8^ V/m.

*r* (Å)	1/*σ*^2^ (Å^−1^)	$\phi_{\hat{r}}^{\alpha }$ (°)	$\eta_{\hat{r}}^{\alpha }$	$\phi_{q}$ (°)	$\eta_{q}^{\alpha }$
5.62 ^a^ (5.50) ^b^ *6.03* ^c^	18.69 (26.50) *15.83*	42.82 (61.29) *68.66*	32.83 (18.39) *30.26*	25.69 (27.57) *19.68*	11.53 (12.63) *9.10*
7.56 (7.16) *6.98*	29.02 (10.31) *6.28*	69.82 (66.53) *70.15*	25.72 (26.17) *36.01*	32.55 (11.53) *28.62*	20.12 (16.93) *22.26*
8.69 (8.53) *8.55*	15.62 (23.19) *21.51*	78.18 (82.31) *80.66*	15.70 (18.63) *19.25*	16.76 (15.92) *17.39*	23.17 (9.13) *16.52*

^a^ The values of the average peak locations and concentration parameters corresponding to the positive direction of the *a*-axis. ^b^ The values of the average peak locations and concentration parameters (in parentheses) corresponding to the positive direction of the *b*-axis. ^c^ The values of the average peak locations and concentration parameters (in italics) corresponding to the positive direction of the *c*-axis.

## Data Availability

The data related to this research can be accessed upon reasonable request via email.

## Supplementary Materials

The following supporting information can be downloaded at: https://www.mdpi.com/article/10.3390/molecules29051159/s1, Figure S1: Molecular structure of TKX-50; Table S1: Crystal data and structural parameters of TKX-50 without the external electric field; Table S2: Atomic coordinates and equivalent temperature factor of TKX-50 without the external electric field; Table S3: Selected bond lengths of the TKX-50 without the external electric field; Table S4: Selected bond angles of the TKX-50 without the external electric field; Table S5: Selected dihedral angles of the TKX-50 without the external electric field; Table S6: Crystal data and structural parameters of TKX-50 under the external electric field; Table S7: Atomic coordinates and equivalent temperature factor of TKX-50 under the external electric field; Table S8: Selected bond lengths of the TKX-50 under the external electric field; Table S9: Selected bond angles of the TKX-50 under the external electric field; Table S10 Selected dihedral angles of the TKX-50 under the external electric field.
