# Supplementary material for: Experimental and Theoretical Investigation of External Electric-Field-Induced Crystallization of TKX-50 from Solution by Finite-Temperature String with Order Parameters as Collective Variables for Ionic Crystals
Source: Molecules. 2024 Mar 5;29(5):1159. doi: 10.3390/molecules29051159 (PMC10934131; doi:10.3390/molecules29051159)
Supplement: Supplementary file 1 [file molecules-29-01159-s001.zip › molecules-2899614-supplementary.pdf]

Supporting Information

Experimental and Theoretical Investigation of External Electric-Field-Induced Crystallization of TKX-50 from Solution by Finite-Temperature String with Order Parameters as Collective Variables for Ionic Crystals

Fude Ren [1,*], Xiaolei Wang [1], Qing Zhang [1], Xiaojun Wang [2], Lingling Chang [1] and Zhiteng Zhang [1]

Figure S1 Molecular structure of TKX-50

Table S1 Crystal data and structural parameters of TKX-50 without the external electric field

Table S2 Atomic coordinates and equivalent temperature factor of TKX-50 without the external electric field

Table S3 Selected bond lengths of the TKX-50 without the external electric field

Table S4 Selected bond angles of the TKX-50 without the external electric field

Table S5 Selected dihedral angles of the TKX-50 without the external electric field

Table S6 Crystal data and structural parameters of TKX-50 under the external electric field

Table S7 Atomic coordinates and equivalent temperature factor of TKX-50 under the external electric field

Table S8 Selected bond lengths of the TKX-50 under the external electric field

Table S9 Selected bond angles of the TKX-50 under the external electric field

Table S10 Selected dihedral angles of the TKX-50 under the external electric field

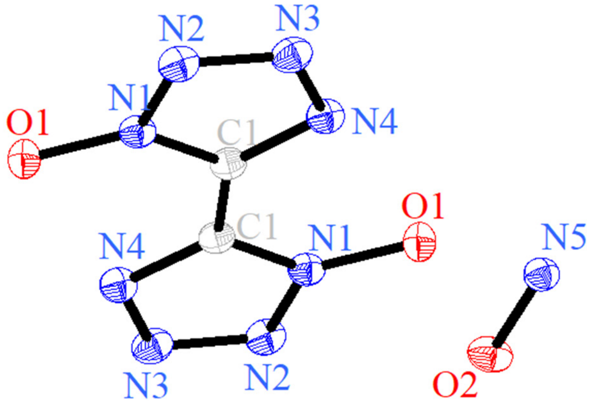

Figure S1. Molecular structure of TKX-50

Table S1 Crystal data and structural parameters of TKX-50 without the external electric field

Parameter	value
Empirical formula	$C_2H_8N_{10}O_4$
Formula weight	236.18
Temperature / K	150
Crystal system	monoclinic
Space group	$P2_1/c$
a / Å	5.4622(2)
b / Å	11.5757(5)
c / Å	6.4887(3)
α / °	90
β / °	95.393(2)
γ / °	90
Volume / Å^3	408.46(3)
Z	2
ρ_{calc} g / cm^3	1.920
μ / mm^{-1}	0.173
F(000)	244.0
Crystal size / mm^3	0.16 × 0.09 × 0.08
Radiation	MoKα (λ = 0.71073)
2Θ range for data collection / °	7.04 to 52.762
Index ranges	? ≤ h ≤ ?, ? ≤ k ≤ ?, ? ≤ l ≤ ?
Reflections collected	830
Independent reflections	830 [R_{int} = ?, R_{sigma} = 0.0319]
Data/restraints/parameters	830/4/82
Goodness-of-fit on F^2	1.098
Final R indexes [I>=2σ (I)]	R_1 = 0.0405, wR_2 = 0.0913
Final R indexes [all data]	R_1 = 0.0505, wR_2 = 0.0968
Largest diff. peak/hole / e Å^{-3}	0.29/-0.25

Table S2 Atomic coordinates and equivalent temperature factor of TKX-50 without the external electric field

Atom	$x/10^4$	$y/10^4$	$z/10^4$	$U(eq)/10^3$
O(1)	2343(2)	4122.5(12)	1807.6(19)	17.6(4)
O(2)	6136(2)	2887.0(12)	1092(2)	20.2(4)
N(1)	2383(3)	4134.5(14)	3852(2)	15.5(4)
N(4)	3279(3)	4444.5(14)	7137(2)	17.2(4)
N(5)	7668(3)	3523.2(14)	-129(2)	15.9(4)
N(2)	674(3)	3593.0(14)	4849(3)	18.4(4)
N(3)	1240(3)	3781.8(14)	6826(3)	19.0(4)
C(1)	3962(3)	4658.5(16)	5261(3)	14.9(4)

Table S3 Selected bond lengths of the TKX-50 without the external electric field

Atom	Length/Å	Atom	Length/Å
O(1)—N(1)	1.325(2)	N(4)—N(3)	1.352(2)
O(2)—N(5)	1.413(2)	N(4)—C(1)	1.329(2)
N(1)—N(2)	1.340(2)	N(2)—N(3)	1.309(2)
N(1)—C(1)	1.342(2)	C(1)—C(1^{1})	1.449(4)

Table S4 Selected bond angles of the TKX-50 without the external electric field

Atom	Angle / °		Angle / °
O(1)-N(1)-N(2)	122.14(15)	N(2)-N(3)-N(4)	110.88(15)
O(1)-N(1)-C(1)	129.51(15)	N(1)-C(1)-C(1^1)	123.7(2)
N(2)-N(1)-C(1)	108.34(16)	N(4)-C(1)-N(1)	108.78(16)
C(1)-N(4)-N(3)	105.50(15)	N(4)-C(1)-C(1^1)	127.5(2)
N(3)-N(2)-N(1)	106.50(15)		

Table S5 Selected dihedral angles of the TKX-50 without the external electric field

Atom	Angle/°	Atom	Angle/°
O(1)-N(1)-N(2)-N(3)	-179.55(15)	N(2)-N(1)-C(1)-C(1^1)	-179.2(2)
O(1)-N(1)-C(1)-N(4)	179.39(16)	N(3)-N(4)-C(1)-N(1)	-0.3(2)
O(1)-N(1)-C(1)-C(1^1)	-0.3(3)	N(3)-N(4)-C(1)-C(1^1)	179.4(2)
N(1)-N(2)-N(3)-N(4)	0.4(2)	C(1)-N(1)-N(2)-N(3)	-0.6(19)
N(2)-N(1)-C(1)-N(4)	0.5(2)	C(1)-N(4)-N(3)-N(2)	-0.1(2)

Table S6 Crystal data and structural parameters of TKX-50 under the external electric field

Parameter	value
Empirical formula	$C_2H_8N_{10}O_4$
Formula weight	236.18
Temperature / K	150
Crystal system	monoclinic
Space group	$P2_1/c$
a / Å	5.4551(3)
b / Å	11.4229(1)
c / Å	6.4822(4)
α / °	90
β / °	95.4130(10)
γ / °	90
Volume / $Å^3$	406.13(3)
Z	2
ρ_{calc} g / cm^3	1.933
μ / mm^{-1}	0.174
F(000)	244.0
Crystal size / mm^3	0.12 × 0.07 × 0.05
Radiation	MoKα (λ = 0.71073)
2Θ range for data collection / °	7.042 to 52.718
Index ranges	? ≤ h ≤ ?, ? ≤ k ≤ ?, ? ≤ l ≤ ?
Reflections collected	827
Independent reflections	827 [R_{int} = ?, R_{sigma} = 0.0395]
Data/restraints/parameters	827/4/89
Goodness-of-fit on F^2	1.139
Final R indexes [I>=2σ (I)]	R_1 = 0.0450, wR_2 = 0.1073
Final R indexes [all data]	R_1 = 0.0569, wR_2 = 0.1143
Largest diff. peak/hole / e $Å^{-3}$	0.30/-0.32

Table S7 Atomic coordinates and equivalent temperature factor of TKX-50 under the external electric field

Atom	$x/10^4$	$y/10^4$	$z/10^4$	$U(eq)/10^3$
O(1)	7656(3)	4121.7(15)	8201(2)	16.7(4)
O(2)	3860(3)	2113.2(15)	3908(3)	18.9(4)
N(5)	2331(4)	1476.7(18)	5129(3)	14.9(5)
N(1)	7618(3)	4135.6(17)	6143(3)	14.3(5)
N(4)	6719(4)	4444.9(18)	2864(3)	16.5(5)
N(2)	9325(4)	3589.9(18)	5154(3)	16.7(5)
N(3)	8755(4)	3780.4(18)	3173(3)	17.9(5)
C(1)	6039(4)	4660(2)	4742(3)	13.8(5)

Table S8 Selected bond lengths of the TKX-50 under the external electric field

Atom	Length/Å	Atom	Length/Å
O(1)—N(1)	1.333(2)	N(4)—N(3)	1.351(3)
O(2)—N(5)	1.412(3)	N(4)—C(1)	1.330(3)
N(1)—N(2)	1.339(3)	N(2)—N(3)	1.312(3)
N(1)—C(1)	1.338(3)	C(1)—C(1^1)	1.446(5)

Table S9 Selected bond angles of the TKX-50 under the external electric field

Atom	Angle / °		Angle / °
O(1)-N(1)-N(2)	121.90(18)	N(2)-N(3)-N(4)	110.92(19)
O(1)-N(1)-C(1)	129.34(19)	N(1)-C(1)-C(1^{1})	124.0(3)
C(1)-N(1)-N(2)	108.7(2)	N(4)-C(1)-N(1)	108.7(2)
C(1)-N(4)-N(3)	105.49(19)	N(4)-C(1)-C(1^{1})	127.3(3)
N(3)-N(2)-N(1)	106.18(19)		

Table S10 Selected dihedral angles of the TKX-50 under the external electric field

Atom	Angle/°	Atom	Angle/°
O(1)-N(1)-N(2)-N(3)	-179.72(19)	N(2)-N(1)-C(1)-C(1^{1})	-179.5(3)
O(1)-N(1)-C(1)-N(4)	179.6(2)	N(3)-N(4)-C(1)-N(1)	-0.2(2)
O(1)-N(1)-C(1)-C(1^{1})	-0.3(4)	N(3)-N(4)-C(1)-C(1^{1})	179.7(3)
N(1)-N(2)-N(3)-N(4)	0.4(2)	C(1)-N(1)-N(2)-N(3)	-0.5(2)
N(2)-N(1)-C(1)-N(4)	0.4(3)	C(1)-N(4)-N(3)-N(2)	-0.1(3)
